# Health Beyond Symptoms: A Qualitative Study on Perceptions and Meanings of Health and Health Promotion among Individuals with Serious Mental Illness in Community Mental Health Settings

**DOI:** 10.1007/s10597-025-01549-7

**Published:** 2025-10-28

**Authors:** Gesa Pult, Fabian Frank

**Affiliations:** 1 Department of Educational Sciences, University of Education, Freiburg, Germany; 2 Department of Social Work, Protestant University of Applied Sciences Freiburg, Freiburg, Germany

**Keywords:** Serious mental illness, Physical health, Community mental health, Health promotion, Qualitative study, Subjective health orientations

## Abstract

Individuals with serious mental illnesses (SMI) face significant health disparities, also affecting physical health. While Community Mental Health (CMH) services primarily support mental recovery, their potential to address the physical health needs of this population remains insufficiently understood. In particular, little is known about how service users conceptualize health and evaluate support efforts - especially with regard to physical well-being. Gaining insight into these perspectives is essential for developing person-centered health promotion strategies that align with users’ lived experiences and contribute to reducing persistent disparities. This qualitative study explored, how individuals with SMI understand health in everyday life, how they perceive the role of CMH professionals in supporting their health, and how they make sense of and respond to health promotion efforts - particularly those targeting physical well-being. Twenty-three qualitative interviews with users of CMH services in Germany were analyzed using a reconstructive, comparative approach that aimed to capture meaning-making processes embedded in everyday social practice. Participants expressed three distinct subjective health orientations: (a) agency-oriented, viewing health as the ability to shape one’s life according to personal goals; (b) stability-oriented, emphasizing inner balance, emotional control, and predictability; and (c) functionality-oriented, focusing on the capacity to manage everyday tasks. These orientations were conceptually distinct and shaped participants’ understanding of health, their attitudes toward CMH services, and their evaluation of health promotion. Health was understood as embedded in daily routines, relationships, and biographical experience. Psychological stability was seen as interconnected and essential for physical well-being. CMH professionals were seen as consistent and trusted partners who promote health through close, everyday relationships and hands-on support. Health promotion and support for physical health was strongly welcomed - if it was voluntary, respectful, and practically integrated into daily life. Health was defined not through symptoms, but as a lived, everyday experience shaped by stability, agency, and biographical context. Health promotion was broadly welcomed when it was voluntary, respectful, and practically embedded in daily life. Although CMH services are primarily focused on mental health, they offer a promising environment for addressing the physical health needs of individuals with SMI.

**Supplementary Information:**

The online version contains supplementary material available at 10.1007/s10597-025-01549-7.

## Background

Individuals with serious mental illnesses (SMI) face significant health disparities, including markedly reduced life expectancy and elevated rates of chronic physical illnesses such as diabetes and cardiovascular diseases (Chan et al., 2022; Druss et al., 2011; Hjorthøj et al., 2017; Wahlbeck et al., 2011; Walker et al., 2015). Multiple, interacting factors contribute to these disparities. For example, side effects of psychotropic medication such as weight gain and metabolic disturbances can contribute to the development of physical illnesses (Bak et al., 2014; Correll et al., 2015; De Hert et al., 2012). In addition, behavioral risk factors, such as smoking, physical inactivity, and unhealthy eating habits, increase the likelihood of preventable diseases like cardiovascular conditions (Cook et al., 2014; Vancampfort et al., 2017). At the same time, structural inequalities – including stigma, fragmented services, and reduced access to timely medical care – further hamper individuals with SMI in attaining good physical health (Grassi & Riba, 2021; Lawrence & Kisely, 2010).In response, international initiatives increasingly emphasize the need for targeted strategies to promote physical health in this population (Firth et al., 2019; Maj, 2009; Stewart, 2015). Health promotion is defined as the process of enabling people to increase control over and improve their health (De Vries et al., 2018; WHO, 1986). This approach shifts the focus from disease treatment to the strengthening of personal capacities, self-determination, and life quality (Huber et al., 2011; Jackson et al., 2005; Kaba-Schönstein, 2018). From this perspective, health is not a static biomedical state but a dynamic and context-sensitive resource for navigating everyday life (Franzkowiak & Hurrelmann, 2022). This aligns with core ideas of the recovery approach, which frames health as a subjective, socially embedded, and empowering process (Dell et al., 2021). Rather than focusing solely on symptom remission, recovery emphasizes personal transformation, community participation, and autonomy in health-related decision-making. Integrating such principles can help make health promotion more meaningful and relevant for individuals with SMI (De Ruysscher et al., 2017; Dell et al., 2021; Piat et al., 2017).

Community Mental Health (CMH) services play a crucial role in promoting recovery, psychosocial functioning, and fostering social inclusion while ensuring continuity of care (Bajraktarov et al., 2020). Delivered by multidisciplinary teams and grounded in everyday life, they are often described as well suited for relational, low-threshold, and context-sensitive approaches to health promotion – that is, approaches based on trust, continuity, and mutual engagement between service users and professionals (Fink-Samnick, 2021; Glasgow et al., 1999; Shaw et al., 2019). While CMH services have traditionally focused on mental and psychosocial support, their potential to promote physical health has received growing attention, especially given the persistent and well-documented disparities faced by SMI. A growing body of evidence supports the relevance of physically oriented interventions, such as exercise, nutritional counseling, and chronic disease prevention, particularly when these are tailored to users’ life contexts, capacities, and motivational needs (Mucheru et al., 2017; Schmitt et al., 2018; Teasdale et al., 2017). However, implementation within CMH services remains limited. Recent qualitative findings suggest that health promotion is most feasible when it builds on users’ everyday routines, social support, and personally meaningful goals (Mucheru et al., 2017; Schmitt et al., 2018; Teasdale et al., 2017).

CMH systems differ significantly across countries. While the UK and the Netherlands have increasingly integrated health promotion into medically anchored CMH frameworks, Germany’s CMH services – understood here as community-based social care, especially assisted living – are primarily psychosocial in nature and situated outside the healthcare system. Their focus lies in fostering social participation and daily functioning despite enduring mental health conditions (Dörner et al., 2019; Simon, 2017). By contrast, in countries such as the UK or the Netherlands, CMH professionals often have more medically oriented roles, which underlines that our findings primarily reflect the German context and should be interpreted with these differences in mind (Sowers et al., 2022). Contemporary policy frameworks increasingly call for holistic, prevention-oriented, and person-centered approaches that bridge mental and physical health, while promoting cross-sectoral integration of health and social care (Firth et al., 2019; WHO, 2013). These developments underline the importance of exploring how health promotion can be meaningfully embedded in community-based support for individuals with SMI, thereby reducing persistent disparities (Firth et al., 2019; Maj, 2009; Stewart, 2015). However, health promotion, especially with regard to physical well-being, remains underdeveloped in the German CMH context (Gühne et al., 2018). This underlines the need to develop context-sensitive, user-informed strategies, which reflect the lived realities and health needs of this population.

This qualitative study responds to this gap - namely, the limited attention to health promotion for individuals with SMI within the German CMH context – by exploring how individuals with SMI conceptualize health in the context of their daily lives, and how they experience and make sense of health promotion efforts within CMH settings, particularly those aimed at improving physical well-being. While mental health promotion focuses on strengthening protective factors and well-being in relation to psychological health, our study addresses the largely neglected area of physical health promotion for individuals with SMI. This focus does not imply a separation of mental and physical health, but responds to the persistent disparities in physical health outcomes in this population. In the German CMH context, where targeted health promotion remains underdeveloped, this makes it particularly important to explore how individuals with SMI perceive existing forms of support and health promotion initiatives. Drawing on the experiences of individuals supported through Germany’s community-based mental health services, the study seeks to generate practice-relevant insights into three core questions:

(1) How do individuals with SMI define what it means to be healthy?

(2) How do individuals with SMI perceive the role of CMH professionals in supporting health?

(3) How do individuals with SMI perceive and interpret the idea of targeted health promotion, and what needs, preferences, and barriers do they associate with it in everyday life?

## Methods

### Study Design

To address these questions, the study employed a qualitative-interpretive design aimed at capturing how individuals with SMI conceptualize and make sense of health health and health promotion within the context of CMH services in Germany. Rooted in a reconstructive research paradigm, the study aimed to capture meaning-making processes embedded in everyday social practice (Helfferich, 2011; Kruse, 2015).

### Participants

The sample included adults diagnosed with SMI who were currently receiving services through Germany’s CMH system. SMI was defined as a long-term, functionally impairing mental disorder, typically within the affective or psychotic spectrum. Individuals experiencing an acute crisis or lacking the capacity to consent were excluded. Participants were recruited in a large German city and two adjacent rural districts. A purposive sampling strategy was applied to ensure an approximately balanced gender distribution and the inclusion of diagnoses commonly represented in CMH settings. Participation was voluntary throughout. Local CMH providers were invited to share information about the study with eligible clients. Interested individuals could then contact the research team directly. Participants received written study information and gave informed consent after being informed about the study’s objectives, their rights as participants, and the professional background of the interviewer, who is a qualified social worker with professional experience in the CMH field. They were assured that participation was voluntary and could be withdrawn at any time. The final sample (*N* = 23) included a diverse range of participants in terms of age, gender, diagnosis, and living situation. Detailed demographic characteristics are presented in the Results section. The study was approved by the ethics committee of the University of Freiburg (reference: 24–1354-S2) and was pre-registered on the Open Science Framework (OSF) (Pult, 2024).

### Data Collection

Between December 2024 and February 2025, 23 qualitative interviews were conducted in person with individuals receiving support from CMH services. Interviews lasted between 25 and 70 min, were audio-recorded and all conducted by the first author. Interviews took place in locations chosen by the participants to ensure comfort and privacy. Most interviews were conducted in participants’ own homes, at CMH service offices, or, in some cases, at a meeting room provided by the Protestant University of Applied Sciences Freiburg. In order to ensure openness and reduce social desirability effects, there was no pre-existing relationship between interviewer and participant prior to the interview. A semi-structured interview format was used, combining open-ended, narrative-oriented introductory questions with thematically focused prompts (Helfferich, 2011). To allow for openness, the core research questions did not explicitly mention ‘physical health’. This enabled participants to introduce dimensions of health most meaningful to them. If physical health was not addressed spontaneously, it was introduced by the interviewer later in the conversation. This approach enabled both narrative depth and analytical comparability across interviews. The interview guide was developed based on the findings of a prior systematic literature review (blinded for review), which identified barriers and facilitators for health promotion among individuals with SMI using the COM-B model (Capability, Opportunity, Motivation – Behavior) as a theoretical framework (Michie et al., 2011). Accordingly, the interview questions were structured to explore participants’ capabilities, opportunities, and motivations with regard to health and health-promoting behaviours. Key interview prompts included:

- “What does being healthy mean to you?”
- “How do you perceive the role of CMH professionals in supporting health?”
- “What do you think about offering targeted health promotion for individuals with SMI?”

### Analysis

All interviews were transcribed verbatim, with all identifying information removed or altered to ensure participant anonymity and confidentiality. The analysis followed the principles of the Integrative Basic Method (Kruse, 2015), developed by German sociologist Kruse (2015). This qualitative approach combines hermeneutic openness with systematic, comparative case analysis and incorporates methodological elements from Grounded Theory (Glaser et al., 2010). This approach is particularly suited to explore latent meaning structures in complex social and biographical contexts. Initial interpretation focused on sequences rich in personal meaning – such as metaphoric expressions, narrative transitions, or reflective passages – using a threefold attentional lens: interactional (e.g., turn-taking, emphasis), structural (e.g., pauses, repairs), and semantic (e.g., choice of words, imagery). These levels were used not as rigid coding schemes but as heuristic prompts to deepen the understanding participants’ meaning-making. Rather than aiming at the construction of fixed typologies, the analysis focused on identifying recurring interpretive orientations, that is, shared logic through which participants make sense of health and illness. These orientations (e.g., autonomy, stability, functionality) emerged from cross-case comparisons and within-case reconstructions and were understood as contextualized patterns of subjective interpretation, shaped by personal biography and lived experience. They are not individual attributes, but interpretive frameworks grounded in lived experience that inform everyday action and expectations around health. Two interdisciplinary peer groups (each involving three researchers) contributed to iterative interpretation and ensured reflexive distance. Throughout the process, a suspensive attitude (Lucius-Hoene & Deppermann, 2002) was maintained to avoid premature closure and to allow participants’ perspectives to unfold within their own frames of reference. All quotations from the interviews were translated from German into English. Minor linguistic adaptations were made to enhance readability without altering meaning.

### Example of Analytical Interpretation

To illustrate the analytic process, the following interview passage is analyzed using the multi-level approach described above. This serves to enhance transparency and demonstrate how meaning was interpreted from the data.

*“For me*,* being healthy means getting up in the morning*,* going to work*,* doing what you have to do. And thank God the medication keeps me from feeling that I actually have a kink up here (points to the head).” (I_16)*.

This passage was interpreted along the three levels of analysis, guided by sensitizing concepts such as understanding of health, positioning, and the use of metaphors:

- **Interactional**: The statement appears early in the interview and serves as a form of self-positioning. The speaker presents himself as someone who defines health primarily in terms of functionality. The fluent articulation, without hesitation or correction, indicates a routine self-description. The accompanying gesture toward the head when mentioning psychological problems reinforces the statement physically and clearly distinguishes between psychological symptoms and outward actions.
- **Syntactic**: The structure is linear, purposeful, and free of digressions: from everyday actions (“getting up… going to work”) to a sense of duty (“doing what you have to do”), and finally to pharmaceutical regulation. The concise, structured formulation underscores a functional view of health, leaving little room for ambivalence or emotional nuance.
- **Semantic**: The phrase “doing what you have to do” refers to an ethos of duty, while “thank God” conveys a certain relief about the stabilizing effect of medication. The metaphor “a kink up here” describes the mental illness in a distanced yet normalizing manner. Notably, there is an absence of relational or emotional references: health appears primarily as a prerequisite for managing daily life, rather than as an expression of subjective well-being.

The excerpt illustrates how health is pragmatically framed as functionality rather than emotional or social well-being. This analysis shows how meanings of health are constituted linguistically and physically throughout the narrative – and how concrete insights into individual orientations toward mental illness and health promotion can be derived from this.

## Results

The analysis of 23 interviews with individuals with SMI identified three distinct subjective health orientations: agency-oriented, stability-oriented, and functionality-oriented. In the following, we present these orientations along with participants’ everyday and biographical understandings of health, the perceived link between psychological and physical well-being, the role of CMH professionals, and views on targeted health promotion.

### Sample

The sample consists of 23 individuals with SMI (12 female, 11 male), aged between 27 and 68 (M = 50.4; MD = 54.5). Participants self-reported schizophrenia (11), affective disorders (7), personality disorders (3) and post-traumatic stress disorder (2). The duration of support from CMH services varied between 1.5 and 26 years, (M = 9.4; MD = 8). The diversity of participants forms the background for the following analysis of subjective understandings of health and the associated individual health orientations. Although the sample included both women and men, no gender-specific differences in health orientations or perspectives on health promotion were observed.

### Health Orientations: Agency, Stability, and Functionality

Participants did not primarily associate health with symptom absence or clinical recovery - an important departure from clinical definitions of health that highlights the relevance of everyday, experiential perspectives.

Instead, three distinct overaching subjective orientations emerged from the comparative-reconstructive analysis, representing different ways of making sense of what means to be healthy. These orientations served as the analytical foundation for the subsequent thematic findings and are closely tied to how individuals approach their daily lives.

The **agency-oriented** group understood health as the ability to shape one’s life in a self-determined and goal-directed way. Health was described as the capacity to pursue personal aspirations and make autonomous decisions aligned with one’s values and future goals:

*“To me*,* health means having goals in life and being able to achieve them.” (I_10)*.

This statement exemplifies how participants explicitly framed health as agency, linking it to the pursuit and realization of personal goals. Here, personal agency was central. Participants in this group viewed health as closely linked to decision-making power and the ability to act. Mental illness was often understood as a disruption or challenge to this capacity.

The **stability-oriented** group defined health primarily in terms of maintaining emotional balance and psychological control. Participants in this group frequently used metaphors – such as “keeping my life under control,” “staying on track,” “maintaining balance,” or “keeping a grip on things” – to express their view of health as a continuous struggle for control and predictability.

*“For me*,* being healthy means getting my life under control.” (I_2)*.

Health, from this perspective, is not given but must be actively preserved through routines, structure, and discipline. Any deviation from that order was perceived as a threat to equilibrium and well-being.

The **functionality-oriented** group expressed a more pragmatic view of health, emphasizing the capacity to manage daily life and fulfill basic responsibilities. Health was described as a minimal condition that enables everyday functioning, rather than a higher-order ideal:

*“Being healthy means getting up in the morning*,* going to work and doing what you have to do.” (I_16)*.

In this perspective, health is not an ideal state, but a minimum level of performance that allows us to “get through the day”. The goal is not growth or balance, but the ability to complete tasks with as few disruptions as possible. Although these orientations were not entirely mutually exclusive, they offer analytically useful distinctions. Each reflects a specific logic through which health was interpreted, shaping participants’ health-related behaviors, needs, and preferences for support throughout the analysis.

### Health as an Everyday and Biographical Experience

Building on the previously identified orientations, this section presents a complementary thematic finding, showing that health is not perceived as an abstract concept, but as a lived experience shaped by the interplay of everyday routines, social relationships, and biographical narratives. As reflected in the orientations, health is not primarily interpreted through medical categories but through concrete life situations and personal meaning-making. Across all three orientations, health was strongly rooted in daily life, anchored in stabilizing routines, social relationships, and concrete living circumstances. For many participants, their own home was a key source of safety and well-being.

*“I feel good yeah. My nice home*,* my siblings are around me […] and I like this” (I_2)*.

Supported housing was widely described as stabilizing, as were work, structured daily activities, or engagement in familiar routines. While participants with an agency-oriented perspective emphasized the role of meaningful relationships in supporting their health, those with a stability-oriented view pointed to the importance of daily routines and structured activities in maintaining a sense of stability and well-being.

*“I need the work at the workshop. That’s important for me.” (I_17)*.

In this way, health becomes a practical space of possibility, enabling participation, order, and the ability to fulfill everyday tasks. In addition to being grounded in everyday life, health was frequently interpreted through biographical reflection.

*“It was hard*,* you know*,* as a kid. My mother didn’t want to take me to the [psychiatric] clinic. We went there*,* had a look*,* and then she said: ‘My daughter’s not going in there.’ So we went back. And somehow*,* I was just left to deal with it on my own. Luckily*,* I still had my brother.”(I_2)*.

Many participants connected their mental health challenges to early life experiences, educational disruptions, or crises in their work life. Such reflections often served to contextualize their current health status and were accompanied by implicit comparisons between “then” and “now.” For some, the moment of diagnosis represented a turning point; for others, it provided a retrospective explanation.

*“I always thought I had to work in the regular job market*,* you know? Like*,* I had to be like everyone else. But I just mentally couldn’t t do it… and then I became suicidal. And that was like a turning point. And where I also started to accept that*,* for me*,* the usual answers in life just don’t work.” (I_7)*.

These narratives highlight how health functions as a narrative resource: it helps to integrate disruptions, reconstruct meaning, and situate oneself within one’s life story. For agency-orientated participants, the focus was on which avenues of self-determination and growth might be (re)opened. Stability-oriented individuals emphasized the need for order and separation from past crises, while those with a pragmatic view described health retrospectively in terms of lost and regained capacity to manage everyday life.

### Psychological Stability as a Prerequisite for Physical Health

Regardless of whether individuals with SMI approached health from an agency-, stability-, or functionality-oriented perspective, psychological stability consistently emerged as a foundational precondition for maintaining physical health. This theme illustrates the strong interdependence between psychological stability and physical well-being, as perceived by participants. When participants were asked to describe their current state of health and, where necessary, to elaborate on aspects of physical health, they consistently referred to episodes of psychological crisis. These were described as turning points in their overall health, often leading to severe neglect of physical well-being. Even the most basic health-related behaviors lost their perceived meaning. Participants reported being unable to translate previously established health-promoting intentions into action during periods of psychological crisis. For example, they described being unable to get out of bed, maintain hygiene, or eat properly.

*“Then I can’t do anything anymore. Just getting up feels like climbing a mountain.” (I_18)*.

Psychological crises were described as disruptive and paralyzing events that interrupt participants’ experience of health and disable their ability to manage daily life. A recurring theme was the unpredictability of such crises, which led to a persistent sense of vulnerability and latent uncertainty.

*“It gets difficult when the psychosis comes*,* then I’m totally out of it*,* and I start neglecting myself. Then the anxiety kicks in*,* and… I withdraw and don’t want to see anyone at all. That’s how it is.” (I_2)*.

Even in more stable phases, the fear of relapse remained present and contributed to what participants described as a “cautious” approach to their own resilience and physical boundaries in everyday life. Physical consequences included both notable weight loss and more frequently rapid weight gain, particularly in the context of psychiatric hospitalization and psychotropic medication. The loss of perceived control over one’s physical state was described as especially distressing. Participants frequently reported that connections between their medication, institutional care, and resulting bodily changes were poorly explained, rarely addressed, or entirely omitted from treatment discussions.

*“I entered the hospital at a normal weight and six months later I was 100 pounds heavier. But I didn’t even know it was because of the medication.” (I_21)*.

Taken together, these findings suggest that psychological stability is not merely one dimension of health, but a necessary foundation for the maintenance of physical health. Across all orientations, participants conveyed that without a basic level of mental stability, it is nearly impossible to sustain or improve physical well-being.

### CMH Services as a Perceived Community of Shared Responsibility

Amidst the tension between psychological strain and the pursuit of stability, participants described CMH not only in terms of organizational offers but above all through their close relationships with one or two trusted professionals. These professionals were experienced as enduring, everyday companions who helped individuals manage health-related challenges, pursue personal goals, and remain capable of acting even during periods of crisis. In this sense, CMH was perceived as a community of shared responsibility, embedded both in stable interpersonal bonds and in the supportive structures of the service organization.

Three core conditions were identified as prerequisites for such health-related conversations: a sense of urgency, sufficient time, and a resilient interpersonal bond. These elements distinguished CMH professionals from other healthcare providers:

*“I trust her[the CMH professional] more than the doctor*,* because he has the power to increase my meds.” (I_6)*.

The perceived role of CMH professionals differed depending on participants’ health orientations: For those with an agency-oriented perspective, CMH relationships served as a space for reflection and goal-setting, often marked by emotional closeness:

*“I always find it surprising how kind she is with me—almost like a friend*,* actually kinder than I am with myself. Yeah*,* she often understands better what’s going on inside me. That’s such a huge relief. I’d say (…) our professional relationship is almost like a friendship.” (I_7)*.

For participants with a stability-oriented perspective, CMH workers were seen as reliable anchors, offering structure and a calm presence that could identify early signs of crisis:

*“If something started to get out of hand*,* she would notice.” (I_17)*.

Functionality-oriented individuals emphasized the practical value of CMH support, such as help with appointments, everyday organization, or simply the reassurance of not being alone in emergencies:

*“If you have a question or a problem […] you can come to him with anything. […] Yeah*,* that’s great.” (I_16)*.

CMH professionals were thus not perceived as conventional experts, but as consistent and attuned partners in care, adapting to individual needs, offering continuity, and fostering conditions in which health and stability become achievable. Their support went beyond singular interventions: they opened spaces for flexible, contextualized assistance rooted in participants’ lived realities.

### Tailored Health Promotion: Absolutely Essential, but not Imposed

When asked about targeted health promotion for individuals with SMI, participants expressed consistent and often enthusiastic support. This section presents their perceptions of such initiatives, particularly those aimed at improving physical health, and highlights the conditions under which they were experienced as meaningful and supportive. Individuals with SMI described such initiatives as “super great”, “urgently needed”, “very meaningful”, and “progressive and totally aligned with me”. These statements reflected more than simple approval – they conveyed a deep sense of validation, relief, and appreciation that their physical health needs were finally being acknowledged and addressed.

Participants’ responses also revealed a strong sense of vulnerability regarding their physical well-being and a shared recognition that these needs are often overlooked, especially in psychiatric services focused primarily on symptom control. Against this backdrop, health promotion was not only welcomed but regarded as an urgently needed form of support.

*“It’s not just that the body affects the soul*,* but also […] the other way around – that you can help the soul through the body.” (I_5)*.

Across all orientations, participants showed interest in health promotion initiatives that addressed both physical and mental wellbeing and were rooted in everyday contexts. Two conditions emerged as essential: voluntary participation and practical relevance – meaning support that addresses the tangible, recurring challenges of service users and fits their lived realities.

*“Don’t force it on us. Like*,* ‘you’ve got to show up for sports now and stuff’ – I don’t like that” (I_17).*

They strongly rejected formats perceived as coercive or based solely on behavioral control. Instead, they emphasized the importance of supportive, dialogical interventions that resonate with their own motivation and are free from stigma. Ideally, health promotion should be enjoyable, meaningful, and pressure-free, something that, in their view, is currently often lacking in existing programs.

*“When I see how one of those rehab exercise groups works*,* that part doesn’t get picked up at all. In the end*,* it’s still all about performance.” (I_5)*.

The perceived value of health promotion was closely tied to the participants’ individual health orientations:

Participants with an agency-oriented perspective viewed health promotion as an opportunity for personal development and self-determination. They expressed a desire to co-design and regarded health behavior as an expression of agency. Stability-oriented individuals appreciated health promotion when it helped them maintain emotional balance, structure, and prevent relapse. Programs were considered valuable if they contributed to a sense of predictability and control. Those with a functionality-oriented perspective were initially more skeptical toward targeted health promotion. They often attributed difficulties with healthy habits to personal failings rather than to illness-related challenges. However, their interest increased notably when programs were clearly linked to supporting everyday functioning and were perceived as practical and relevant.

Participants also referred to health-related services they had already encountered in CMH settings, such as cooking groups, individual conversations about nutrition, physical activity, or bodily changes. These were generally evaluated positively.

*“My best friend always says that (CMH provider) is my second home—and that’s what it is for me: a place where I can just be how I am in that moment. The CMH offers are the best thing that ever happened to me.” (I_10)*.

Health promotion was not only welcomed by the participants but experienced as necessary, on the condition that it was delivered respectfully, addressed relevant personal concerns, and was embedded in a trusted support relationship. When these conditions were met, participants perceived health promotion as a meaningful contribution to their autonomy and quality of life.

## Discussion

This study explored how 23 individuals with SMI, supported by Germany’s CMH system, define what it means to be healthy, how they perceive the role of CMH professionals in supporting their health, and how they evaluate current health promotion efforts, particularly in light of ongoing recommendations for targeted interventions for this population. Particular attention was given to participants’ needs, preferences, and perceived barriers, especially concerning physical health. Three distinct health orientations emerged: agency-oriented, stability-oriented, and functionality-oriented. These health orientations informed not only how participants defined health, but also how they perceived CMH professionals and evaluated health promotion, while the other result sections highlight complementary thematic dimensions that add further depth to these overarching frameworks.

Health was described not in biomedical terms, but as a lived experience grounded in routines, emotional resilience, and biography. Psychological crises were seen as key barriers to physical health, while CMH professionals were valued as stabilizing, relational partners in a shared responsibility.

Health promotion was widely welcomed, especially when respectful, voluntary, and integrated into daily life. Participants called for approaches that connect mental and physical health and support autonomy, structure, or everyday functioning.

### Methodological Reflections and Limitations

A key strength of this study lies in its qualitative-interpretive design, grounded in a reconstructive research paradigm. Through open-ended interviews and systematic case comparison, it explored how individuals with SMI make sense of health and health promotion in the context of their everyday lives. This approach allowed for the identification of shared interpretive patterns while preserving the specificity of each participant’s biographical and lived experience. The sample size (*n* = 23) aligns with comparable qualitative studies and provided a solid basis for reconstructing cross-case insights grounded in individual perspectives.

However, some limitations must be acknowledged. The study took place within the German CMH context, which emphasizes psychosocial support over clinical treatment, potentially limiting transferability. A purposive sampling strategy was used to ensure diversity in gender and diagnoses typically represented in CMH settings. Nevertheless, self-selection bias cannot be ruled out: participants who volunteered may have been more motivated or more interested in health-related topics than those who declined. This limitation should be considered when interpreting the findings.

Despite these limitations, the study offers important insights into how qualitative methods can uncover experiential meanings of health and inform more context-sensitive, person-centered interventions, also experientially meaningful and aligned with the everyday realities of individuals living with SMI.

### Incorporating Individual Interpretations of Health

The analysis identified three recurring distinctive health orientations among individuals with SMI: an agency-oriented view of health as the ability to shape one’s life, a stability-oriented understanding focused on maintaining inner balance and control, and a functionality-oriented perspective that defines health as a prerequisite for managing everyday tasks. These orientations are not fixed typologies but represent interpretive logics rooted in personal biographies, coping experiences, and psychosocial contexts. They offer a nuanced understanding of how “being healthy” is subjectively grounded beyond medical categories, aligning with broader definitions of health and recovery (De Ruysscher et al., 2017; Franzkowiak & Hurrelmann, 2022; Huber et al., 2011; Piat et al., 2017) and connecting with prior work on illness perceptions and recovery-oriented care (Badura, 1985; Büchi & Buddeberg, 2004; Kiemen et al., 2020).

The orientations can be understood as narrative structures through which individuals situate themselves, their illness, and their everyday health practices. They thus provide a foundation for needs-based support and for the design of health promotion strategies: understanding these orientations helps explain why some interventions are experienced as helpful, while others may be perceived as overwhelming or irrelevant. For example, individuals with an agency orientation may benefit from participatory formats such as personalized coaching or peer-led programs, those with a stability orientation from structured, routine-based offers (e.g. weekly walking groups, scheduled health check-ins), and those with a functionality orientation from low-threshold, pragmatic support (e.g. meal planning, appointment reminders, practical tips for daily physical activity).

Such differentiation supports a shift away from standardized programming toward dialogical, person-centered practices that are responsive to individual meaning systems. This perspective is consistent with recovery-oriented approaches that emphasize participatory and co-designed interventions (De Ruysscher et al., 2017; Piat et al., 2017), the value of structured offers for stability (Slade, 2010; Topor et al., 2011), and evidence for low-threshold, pragmatic support tailored to everyday functioning (Mucheru et al., 2017; Teasdale et al., 2017). In research contexts, these orientations may also serve as an analytical framework for reconstructing how individuals engage with health-related practices over time. Recognizing these interpretive logics highlights individuals with SMI not as passive recipients of care, but as active meaning-makers whose perspectives are essential for developing sustainable, acceptable, and personalized interventions.

### Rethinking Health in the Context of Everyday Life

Rather than defining health as the absence of symptoms or clinical stability, participants described it as a lived experience embedded in daily routines, emotional balance, biographical continuity, and social participation. This perspective challenges biomedical reductionism and aligns with holistic, life-world-centered understandings of health (Franzkowiak & Hurrelmann, 2022; Huber et al., 2011). Participants emphasized the interdependence of mental and physical well-being, the stabilizing role of daily structure, and the importance of supportive relationships. Despite multiple challenges, they showed strong motivation to sustain or improve their health – including physical well-being. This finding contradicts assumptions in the literature that depict individuals with SMI as disengaged or apathetic (Topor et al., 2011). On the contrary, they actively interpreted and negotiated their health in light of symptoms, medication side effects, social stressors, and structural constraints. Health thus emerges as a meaningful and manageable resource, consistent with recovery-oriented principles emphasizing autonomy, participation, and subjective definitions of well-being (De Ruysscher et al., 2017; Piat et al., 2017).

This departure from clinical notions of health as symptom remission represents a central contribution of our study, underscoring the need for strategies that build on experiential and everyday understandings of well-being. Health promotion should not assume a singular model of “healthy behavior” but flexibly respond to individuals’ lived priorities and capabilities. For example, initiatives may focus less on generalized goals (such as weight loss or fitness targets) and more on personalized support, such as reestablishing morning routines, coping with fatigue, or managing medication side effects. Embedded in daily life through small, achievable steps and co-developed with service users, health promotion becomes not a normative goal but a shared process of meaning-making and adaptation.

### Community Mental Health as a Suitable Setting for Health Promotion

The findings underscore the potential of CMH services as meaningful settings for health promotion among individuals with SMI – not despite, but because of their relational, everyday-oriented approach to care (Bajraktarov et al., 2020; Fink-Samnick, 2021; Gahleitner, 2017; Steimle et al., 2025). Our findings also indicate that service users rarely differentiate between CMH as an organization and CMH professionals. In practice, CMH is experienced primarily through enduring relationships with trusted staff, while organizational offers provide the supportive framework for these relationships. CMH professionals were not described as distant experts, but as consistent allies who help maintain routines, provide orientation during crises, and recognize small but significant progress. Health promotion thus becomes a co-constructed, dialogical process embedded in lived realities. This applied across all health orientations: agency-oriented participants experienced CMH professionals as a space for reflection and growth; stability-oriented participants valued structure and predictability; and functionality-oriented participants highlighted practical support in everyday tasks.

These insights show that health promotion is most meaningful when dialogical rather than prescriptive, supportive rather than directive, and individually relevant. This perspective also aligns with the COM-B model (Michie et al., 2011), which conceptualizes health behavior as the result of Capability, Opportunity, and Motivation. The interview guide was explicitly informed by this framework, allowing exploration of how individuals perceive their capabilities, opportunities, and motivational dynamics. CMH services and their professionals can enhance capability through dialogical support, provide opportunity through relational continuity, and foster motivation by validating concerns and building on personally meaningful goals.

Although CMH services have traditionally focused on psychosocial support, participants emphasized the deep interconnection between mental and physical health. Support for physical health was often described as insufficient, highlighting an untapped opportunity for CMH to expand toward holistic and integrated models. Such an expansion does not require abandoning core principles, but applying them to physical health needs. By tailoring interventions to individual preferences, life contexts, and orientations, CMH professionals can foster sustainable engagement without standardized or coercive approaches.

Finally, the results resonate with contemporary European and German policy strategies calling for more integrated, recovery-oriented, and person-centered care (Gühne et al., 2018; Simon, 2017; WHO, 2013). Strong user support for respectful and everyday-oriented health promotion corresponds closely with current European recommendations to strengthen person-centered and intersectoral strategies. At the national level, this highlights the untapped potential of German CMH services to expand their scope in line with ongoing debates on integrated care, thereby helping to reduce persistent physical health disparities among individuals with SMI.

### Health Promotion as Recognition and Enabling of Choice

The findings indicate that health promotion is experienced as supportive and meaningful by individuals with SMI when it is voluntary, respectful, and grounded in everyday life. Emotional safety, relevance to real-life contexts, and the experience of being taken seriously were described as essential preconditions for engagement. Under these conditions, health promotion was not seen merely as behavior change, but as recognition of individual needs, perspectives, and self-concepts (Davidson, 2021; Topor et al., 2011, 2018).

This view aligns with the Capability Approach (Altgeld & Bittlingmayer, 2017; Nussbaum, 2011; Sen, 2001), which emphasizes not only access to resources but the freedom to transform them into personally meaningful ways of living. In this context, interventions should be compatible with individual life circumstances, motivational states, and health orientations (Oyserman et al., 2007; Super et al., 2015). Health-related behavior should thus be understood within broader frameworks of biography, meaning-making, and social relationships (Slade, 2010).

Importantly, this also applies to physical health – a domain often neglected in mental health care. Participants stressed that their physical well-being was deeply interconnected with emotional stability, daily functioning, and self-worth. Promoting physical health was therefore not perceived as a separate task, but as a relational and empowering practice. Rather than standardized behavior modification, health promotion should be understood as a lifelong process that expands meaningful options and enables individuals to pursue health in ways consistent with their values and lived experiences. This perspective is consistent with international recommendations for recovery-oriented and participatory approaches in psychosocial care (Laudet & Humphreys, 2013; WHO, 2013). From this angle, health promotion is not only about preventing risk or encouraging compliance but can serve as a pathway toward self-determination, social participation, and strengthened individual agency.

While this study focused primarily on physical health promotion, it is important to note that health promotion and mental health promotion share many underlying principles, particularly their grounding in everyday life rather than being confined to services (Bajraktarov et al., 2020; Firth et al., 2019; WHO, 2013). Strengthening the connections between services and daily contexts is therefore a key development area across countries. From a policy perspective, intersectoral collaboration between health care, social services, and community-based resources represents a promising avenue to enhance integrated and sustainable health promotion for individuals with SMI.

## Conclusion: Health Beyond Symptoms

This study shows that individuals with SMI do not primarily define health through clinical symptoms, but as a situated capacity: to remain emotionally stable, act meaningfully, and manage daily life. Health was described as embedded in routines, relationships, and biographical experiences—an understanding that challenges biomedical framings and calls for more person-centered approaches.

Participants strongly welcomed health promotion, particularly when it was voluntary, respectful, and integrated into daily life. Psychological stability was seen as a crucial foundation for engaging in health-promoting behaviors, and support for physical health as especially relevant yet often overlooked in CMH practice. Importantly, health promotion should be tailored to individuals’ health orientations—whether centered on agency, stability, or functionality. When aligned with these lived frameworks, support was experienced as empowering rather than directive.

Although CMH services are primarily designed to promote psychosocial inclusion, our findings highlight their untapped potential to also address physical health needs. This recommendation resonates with research on recovery-oriented and participatory approaches (De Ruysscher et al., 2017; Piat et al., 2017), the importance of structured and relational support (Gahleitner, 2017; Slade, 2010; Steimle et al., 2025; Topor et al., 2018), and evidence on the effectiveness of adapted lifestyle interventions (Mucheru et al., 2017; Schmitt et al., 2018; Teasdale et al., 2017). By aligning with current European and national policy strategies (Gühne et al., 2018; Simon, 2017; WHO, 2013), CMH services can play a pivotal role in advancing integrated and equity-oriented health promotion for individuals with SMI.

Ultimately, health promotion in CMH settings should begin with listening: it must be grounded in the lived experiences of users, enable individualized engagement, and foster recognition, agency, and participation in everyday life.

## Supplementary Information

Below is the link to the electronic supplementary material.

Supplementary Material 1

## Data Availability

No datasets were generated or analysed during the current study.
