# Supplementary material for: Health Beyond Symptoms: A Qualitative Study on Perceptions and Meanings of Health and Health Promotion among Individuals with Serious Mental Illness in Community Mental Health Settings
Source: Community Ment Health J. 2025 Oct 28;62(3):571–83. doi: 10.1007/s10597-025-01549-7 (PMC12963103; doi:10.1007/s10597-025-01549-7)
Supplement: Supplementary file 1 — Supplementary Material 1 [file 10597_2025_1549_MOESM1_ESM.pdf]

Leitfadengestütztes Einzelinterview mit Personen, die aufgrund einer schweren psychischen Erkrankung Assistenzleistung zur Sozialen Teilhabe (früher: ambulant betreutes Wohnen)erhalten

Der Leitfaden orientiert sich am COM-B Modell für Behavior Change, als Rahmung für die Ergebnisdarstellung von: *Barriers and Facilitators to Health Behavior Change: Perspectives of Individuals with Serious Mental Illness in Health-Promoting Interventions – A Systematic Review of Qualitative Evidence,* verwendet wurde.
Die wesentlichen Ergebnisse waren:

| | |
|---|---|
| Capabiltiy | Desire to improve physical health |
| Motivation | Motivation to be/ to stay healthy, difficulty to maintain health behavior change in periods of increased psychological symptoms |
| Opportunity | Appreciate social support and networking, easy to understand learning content, practical lessons, and awareness of limited financial situations |

**Für den Fall, dass das Gespräch Sie emotional belastet, wer steht für ein entlastendes Gespräch zur Verfügung?**

| Leitfaden Interview | | | |
|---|---|---|---|
| *Frageblöcke* | *Erzählaufforderung* | *Nachfragen* | *Aufrechterhaltungsfragen* |
| Ice-Breaker | Vielen Dank, dass Sie heute hier sind!<br>Vielleicht bevor wir „richtig“ anfangen würde ich mich freuen, wenn Sie ganz kurz etwas dazu sagen würden, wie kam es denn, dass Sie heute mit mir sprechen? | | |
| **Intro** | | | |
| Gesundheit | Vielen Dank, dann würde ich jetzt mal mit dem Interview beginnen:<br>- Über Gesundheit wird ja viel gesprochen und mich würde ganz zu Anfang mal interessieren: Was verstehen Sie denn unter „gesund sein“? | - Mit wem tauschen Sie sich den vielleicht über gesundheitliche Themen so aus?<br>- Wie würden Sie die Abwesenheit von Gesundheit beschreiben?<br>- Was ist Krankheit für Sie?<br>- worüber sprechen Sie dann? | - Der Punkt ____ ist für mich besonders interessant. Können Sie mir noch etwas mehr darüber berichten?<br>- Können Sie hierfür ein Beispiel benennen?<br>- Und dann, wie ging es weiter? |

| | | | |
|---|---|---|---|
| | - Wie nehmen Sie das wahr: Welche Rolle spielt denn das Thema Gesundheit in Ihrer Betreuung (abW/sbW/Gastfamilie)? | | - |
| **I Capability** | | | |
| körperliche Gesundheit | Jetzt haben wir über Gesundheit und die Rolle dieses Themas hier beim ambulant betreuten Wohnen gesprochen.<br>- Wie würden Sie denn selbst Ihre aktuelle gesundheitliche Situation beschreiben? | - Beim Thema Gesundheit interessiert mich ganz besonders die körperliche Gesundheit. Würden Sie mir mal sagen, wie sie die so bei sich wahrnehmen? | |
| **II Motivation** | | | |
| Veränderun gsmotivatio nen | - Gibt es denn generell etwas, was Sie gerne verändern wollen und wenn ja,  was wäre das? | - Wenn Sie Ihre körperliche Gesundheit so beschreiben. Wie geht es Ihnen damit?<br>- | |
| **III Opportunity** | | | |

| | | | |
|---|---|---|---|
| Veränderungsermöglichungen | Menschen mit psychischen Erkrankungen sind deutlich häufiger von körperlichen Erkrankungen betroffen. Es wird deshalb empfohlen, zukünftig Maßnahmen anzubieten, die eine gesundheitsfördernde Lebensweise unterstützen.<br>- Wie denken Sie darüber? | - Was glauben Sie könnte denn dazu beitragen, dass Sie in Zukunft körperlich gesund bleiben/ gesünder werden?<br>- Meinen Sie, dass körperlich gesund zu bleiben für Menschen mit psychischen Erkrankungen vielleicht noch schwieriger ist, wenn ja, weshalb?<br>- Was könnte da helfen?<br>- Könnten Sie ein Beispiel nennen, wo sie versucht haben, an ihrer körperlichen Gesundheit etwas zu ändern ?<br>- Was hat Ihnen da geholfen oder war vielleicht besonders schwer?<br>- Wie holen Sie sich generell Informationen zu gesundheitlichen Themen? | |
| **IV Gesundheitsangebote** | | | |
| | - Jetzt habe ich viel über Ihre Einschätzung zu gesund sein und körperlicher Gesundheit von Ihnen erfahren. Vielen Dank dafür.<br>- Am Schluss habe ich jetzt noch ein paar kurze und ganz konkrete Fragen mitgebracht:<br>- Würden Sie allgemein mehr über Erfahren über Themen, die mit Gesundheit zu tun haben? | - Gerne können Sie auch weitere Vorschläge machen, die Ihnen in dem Zusammenhang vielleicht wichtig sind. | |

| | | | |
|---|---|---|---|
| | - Würden Sie allgemein gerne mehr erfahren über Zusammenhängen zwischen körperlicher und psychischer Gesundheit?<br>- Würden Sie wünschen, sich zu solchen Themen regelmäßig mit anderen auszutauschen?<br>- Würden Sie gerne mit anderen auch ganz konkret etwas für ihre körperliche Gesundheit tun wollen, z..B. zusammen spazieren zu gehen oder zu kochen und zu essen?<br>- Mal angenommen, es gäbe hier über das ambulant betreute Wohnen ein Angebot, das Sie regelmäßig besuchen könnten, und wo es um das Thema Gesundheit ginge. Würde Sie das interessieren? | | |
| **V Outro** | | | |
| | - Vielen Dank nochmal für Ihre Antworten. Wir wären von meiner Seite aus am Ende des Interviews angelangt. Ist von ihrer Seite aus noch etwas offen, oder möchten sie noch etwas ergänzen oder vervollständigen?<br>- Wie fühlen Sie sich? Wie gehen sie aus dieser Situation? | | |
